# Pycnogenol Attenuates the Release of Proinflammatory Cytokines and Expression of Perilipin 2 in Lipopolysaccharide-Stimulated Microglia in Part via Inhibition of NF-κB and AP-1 Activation

**DOI:** 10.1371/journal.pone.0137837

**Published:** 2015-09-14

**Authors:** Bin Fan, Sai-Hong Dun, Jian-Qiu Gu, Yang Guo, Shoichiro Ikuyama

**Affiliations:** 1 Department of Neurology, Shengjing Hospital, China Medical University, Shenyang, 110004, P. R. China; 2 Department of Endocrinology and Metabolism, First Affiliated Hospital, P. R. China Medical University, Shenyang, 110001, P. R. China; 3 Department of Clinical Investigation & Department of Endocrine, Metabolic and Rheumatic Diseases, Oita San-ai Medical center, Oita, 870–1151, Japan; Stony Brook University, UNITED STATES

## Abstract

Over activation of microglia results in the production of proinflammatory agents that have been implicated in various brain diseases. Pycnogenol is a patented extract from French maritime pine bark (Pinus pinaster Aiton) with strong antioxidant and anti-inflammatory potency. The present study investigated whether pycnogenol may be associated with the production of proinflammatory mediators in lipopolysaccharide-stimulated BV2 (mouse-derived) microglia. It was found that pycnogenol treatment was dose-dependently associated with significantly less release of nitricoxide (NO), TNF-α, IL-6 and IL-1β, and lower levels of intercellular adhesion molecule1 (ICAM-1) and perilipin 2 (PLIN2). Furthermore, this effect was replicated in primary brain microglia. Levels of inducible NO synthase mRNA and protein were attenuated, whereas there was no change in the production of the anti-inflammatory cytokine IL-10. Further evidence indicated that pycnogenol treatment led to the suppression of NF-κB activation through inhibition of p65 translocation into the nucleus and inhibited DNA binding of AP-1, suggesting that these proinflammatory factors are associated with NF-κB and AP-1. We conclude that pycnogenol exerts anti-inflammatory effects through inhibition of the NF-κB and AP-1pathway, and may be useful as a therapeutic agent in the prevention of diseases caused by over activation of microglia.

## Introduction

Microglia cells are resident innate immune cells in the central nervous system, functionally similar to macrophages. The activation of microglia has been observed in various brain diseases and can be induced by lipopolysaccharides (LPSs), β-amyloid and interferon (IFN)-γ[1–4]. An upregulated response or over-activation of microglia can lead to the release of a variety of pro-inflammatory mediators that are potentially neurotoxic. These mediators include nitricoxide (NO), tumor necrosis factor (TNF)-α, interleukin (IL)-1β, IL-6, and intercellular adhesion molecule-1 (ICAM-1)[5–7], which have been implicated in brain diseases such as trauma, seizures, ischemia and Alzheimer’s disease [4]. Suppression of microglia activation, and thus the production of these proinflammatory mediators could be an important therapeutic strategy.

It is known that members of the perilipin (PLIN) family of proteins function in intracellular lipid droplet formation, which is reportedly involved in the activation of LPS-stimulated microglia [8]. These members include PLIN1, PLIN2 (adipose differentiation-related protein, ADRP), PLIN3 (47 kDa tail interacting protein, TIP47), PLIN4 (S3-12), and PLIN5 (oxidative tissue-enriched PAT protein, OXPAT)[9–11]. Particularly, PLIN2 is a prominent lipid droplet-associated protein that is ubiquitously expressed in a variety of cells, predominantly in macrophages and foam cells at the site of atherosclerotic lesions [12]. Additionally, genetic abrogation of PLIN2 prevents atherosclerosis formation and hepatic steatosis [13, 14]. It was recently reported that enhanced expression of PLIN2 and lipid droplet formation are involved in microglia activation [8]. We previously demonstrated that macrophages activated by inflammatory stimuli were associated with enhanced PLIN2 expression [15]. To date, however, little is known about the factors that regulate the PLIN2 gene in microglia.

There is evidence that the transcription factors NF-κB (nuclear factor kappa-light-chain-enhancer of activated B cells) and AP-1 (activator protein-1) are positively involved in the LPS-induced inflammatory response of microglia [16–18]. Thus, inhibition of NF-κB and AP-1 activity may suppress the activity of microglia. We searched among a variety of widely used dietary supplements for compounds with few side effects that may suppress NF-κB and AP-1 activity and the subsequent production of proinflammatory mediators. Such inhibitors might prevent the development of diseases caused by activation of microglia. In the present study, we focused on pycnogenol (PYC), a patented extract of French maritime pine bark (Pinus pinaster Aiton) with strong antioxidant and anti-inflammatory potency in vitro perfused organs and in vivo renal ischemia/referfusion model [19].

We previously showed that PYC treatment led to the suppressed expression of proinflammatory cytokines such as IL-6, IL-1α and IFN-β in LPS-stimulated RAW264.7 (mouse macrophage) cells through inhibition of AP-1 and NF-κB activity, without affecting their DNA-binding function [15]. More recently, we showed that PYC inhibited expression of PLIN2 and lipid accumulation in liver cells, by facilitating degradation of ADRP (or PLIN2) mRNA [20]. Based on these studies, PYC appears to have multiple functions, especially potent inhibition of NF-κB and AP-1activity.

In the present study, we examined the effects of PYC on LPS-stimulated microglia activation and their release of proinflammatory mediators. Since both NF-κB and AP-1 pathways participate in the regulation of inflammatory response in microglial cells, both pathways were examined as possible underlying molecular mechanism.

## Materials and Methods

The experimental protocol was established, according to the ethical guidelines of the Helsinki Declaration and was approved by the Ethics Committee of Shengjing Hospital, Medical University, China.

### Materials

Reagents and suppliers were listed as follows: LPS (from Escherichia coli 0128: B12, Product No. L2755), pyrrolidinedithiocarbamate (PDTC) was purchased from Sigma (St. Louis, MO). PYC was generously provided by Horphag Research (Geneva, Switzerland). Dulbecco’s modified Eagle’s medium (DMEM) containing L-arginine (200 mg/L) and fetal bovine serum (FBS), and other tissue culture reagents were purchased from Gibco (Invitrogen, Carlsbad, CA). Griess reagent and methyl thiazolyl tetrazolium (MTT) was from Sino-American Biotechnology (Beijing, P. R. China). Rabbit anti-mouse inducible NO synthase (iNOS; sc-8309), p65 (sc-372), nucleolin (sc-13057), and glyceraldehyde-3-phosphate dehydrogenase (GAPDH; sc-25778) antibodies were supplied by Santa Cruz Biotechnology (Santa Cruz, CA, USA). Antibody to PLIN2 (GP47) was purchased from PROGEN Biotechnik (Heidelberg, Germany). Iba-1 antibody was obtained from Wako (Osaka, Japan). Peroxidase-labeled goat anti-rabbit antibody (bgt-0718) was obtained from Amersham (Arlington Heights, IL, USA). Specific ELISA kits for detection of mouse TNF-α (MTA00B), IL-6 (M6000B), IL-1β (MLB00C), and IL-10 (#1000) were purchased from R&D systems (Minneapolis, MN, USA). Synthesized oligonucleotide was purchased from Roche Diagnostics (Tokyo, Japan). Deoxycytidine triphosphate (dCTP), α-^32^P and γ-^32^P ATP were purchased from GE Healthcare (Buckinghamshire, UK). Other chemicals were purchased from Sigma.

### Cell culture

The BV2 cells were purchased from cell resource center, IBMS, CAMS/PUMC (Beijing, P. R. China). The murine BV2 microglial cells were cultured in DMEM supplemented with 10% FBS, 100 U/mL penicillin, and 100 mg/mL streptomycin, and maintained in a humidified incubator with 5% CO_2_. Generation and culture conditions of mouse primary brain microglia have been described previously [21]. Briefly, cerebral cortices were isolated from 1 day old C57BL/6J mice and were dissociated, digested with 0.25% Trypsin-EDTA solution for 20 min at 37°C. The cells were pelleted at 200g for 5 min, re-suspended and then passed through a 70 μm pore filter. The cells were re-suspended in DMEM nutrient mixture supplemented with 10% FBS, penicillin (100 U/ml) and streptomycin (100 mg/ml), and then, were pelleted and re-suspended. The cells were seeded in 75 cm^2^ flasks for 18 days. Primary microglia cells were separated from mixed glial cells by vigorous shaking for 4 hours at 200 rpm at 37°C. The separated cells were cultured in the medium and seeded into 24-well plates at a density of 5×10^5^ cells/well. One hour later, non-adherent cells were removed. After 2–3 days of culture, the cells were prepared for treatments. The purity of the primary microglia cells were more than 95% as determined by Iba-1 staining.

### Nitrite assays (Griess assay)

NO levels in culture supernatants were measured by Griess reaction. The cells were seeded in 24-well plates (5 × 10^5^ cells/mL) and incubated with PYC (10, 25, 50 μg/mL) or DMSO (vehicle) for 1 h before addition of LPS (500ng/mL) for 24h. After treatments, 100 μL of conditioned culture medium from each sample was mixed with the same volume of the Griess reagent (i.e., 1% sulfanilamide/0.1% N-[1-naphthyl]-ethylenediamine dihydrochloride/2.5% H_3_PO_4_). The concentration of NO was determined by measuring its absorbance at 540 nm with a 96-well microplate spectrophotometer (Labsystems, Franklin, MA, USA). The nitrite concentration was calculated with reference to a standard curve for sodium nitrite generated by known concentrations.

### Cytotoxicity assay

Cell viability was evaluated using the MTT reduction assay. Briefly, cells (5 × 10^4^ cells/mL) were seeded into 96-well plates overnight. PYC was incubated at various concentrations or DMSO vehicle for 1 h (10, 25, 50 μg/mL). Then LPS (500 ng/mL) was added and incubated with the cells for 24h. After treatments, the medium was removed and the cells were incubated with 0.5 mg/mL of MTT solution. After incubation in 5% CO_2_ for 3 h at 37°C, the supernatant was removed and the formation of formazan crystals was measured at 540 nm with a microplate reader.

### Real-time PCR

The mRNA levels of iNOS, ICAM-1 and PLIN2 were measured by Real-time PCR. Cells were incubated with PYC (10, 25, 50 μg/mL) or DMSO (vehicle) for 1 h, LPS (500ng/mL) was then added and incubated for 24h. Total RNA was extracted using TriReagent (Sigma-Aldrich, St. Louis, MO), in accordance with the manufacturer’s instructions. Single-stranded cDNA was synthesized with a ReverTra Ace-α kit (Toyobo, FSK-101, Osaka, Japan) using 0.5 μg total RNA.

Real-time PCR was performed with a SYBR Green method using an ABI prism 7500 Sequence Detection System (Applied Biosystems, Foster City, CA) in a 25-μL reactionvolume (12.5 μL of 2× iQ SYBR Green supermix (Bio-Rad, #170–8882, Hercules, CA), 320 nM for each primer, and 5 μL of 1:20 diluted cDNA). The primer sequences were: iNOS sense, AATGGCAACATCAGGTCGGCCATCACT, and antisense, GCTG-TGTGTCACAGAAGTCTCGAACTC; ICAM-1 sense, TGCGTTTTGGAGCTAGCGGACCA, antisense, CGAGGACCATACAGCACGTGCCAG; PLIN2 sense, CTGTCTACCAAGCTCTGCTC, and antisense, CGATGCTTCTCTTCCACTCC; GAPDH sense, ACCACAGTCCATGCCATCAC, and antisense, TCCACCACCCTGTT-GCTTA. PCR efficiencies for all reactions were > 0.90. Quantitative PCR results were expressed as relative induction fold relative to the housekeeping gene GAPDH.

### Western blot analysis

Protein levels of iNOS, PLIN2 and nuclear protein NF-κB p65 were tested by using Western blot analysis, as described previously [20]. BV2 cells were treated with PYC at various concentrations or DMSO (vehicle) for 1h (10, 25, 50 μg/mL). LPS (500 ng/mL) was then added and further incubated for 24 h. The cells were harvested using radioimmunoprecipitation (RIPA) lysis buffer containing 50 mM Tris-HCl (pH 7.4), 150 mM NaCl, 1% sodium deoxycholate, 1% NP-40, 1 mM phenylmethylsulfonyl fluoride (PMSF), and 1 mM ethylenediaminetetraacetic acid (EDTA).

In a parallel experiment, the nuclear protein was prepared using lysis buffer (10 mM Tris-Cl pH 7.4, 3 mM CaCl_2_, 2 mM MgCl_2_, 0.5 mM dithiothreitol, 0.5 mM PMSF, and protease inhibitors) for 15 min at 4°C. After centrifuging and washing the pellet, ice-cold hypertonic extraction buffer containing 20 mM hydroxyethyl piperazinee thanesulfonic acid (HEPES; pH 7.1), 25% glycerol, 420 mM NaCl, 0.2 mM EDTA, 1.5 mM MgCl_2_, 0.5 mM dithiothreitol, and protease inhibitors was added and the samples were incubated at 4°C for 30 min with constant shaking. The nuclear protein extracts were isolated by centrifugation at 12000×g for 30 min.

The proteins were heated at 95°C for 5 min, resolved via 10% SDS-PAGE and electroblotted onto a polyvinylidene difluoride membrane (MSI, Westborough, MA) for 1 h at 100 V with a Western blot apparatus (Bio-Rad, Hercules, CA) in Tris-glycine transfer buffer containing 25 mM Tris, 192 mM glycine, 20% methanoland 0.1% SDS.

The membranes were blocked for 1 h at room temperature with 5% non-fat milk in phosphate-buffered saline (PBS) containing 0.1% Tween 20 (PBS-Tween 20). The membranes were then incubated sequentially with antibodies against GAPDH (1:500), iNOS (1:1200), PLIN2 (1:1000) or NF-κB p65 (1: 500) for 1 h. After washing four times (5 min each) with PBS-Tween 20, the membranes were incubated with secondary IgG-horseradish peroxidase conjugate antibodies (1:2000) diluted in PBS-Tween 20 (0.08 μg/mL) for 1 h, and then washed in buffers described as above. The immunoreactivity was detected by an enhanced chemiluminescence technique.

The levels of protein were quantified using the NIH image processing and analysis program. Three independent experiments were carried out and a representative result is shown.

### Enzyme-linked immunosorbent assay (ELISA)

Protein levels of TNF-α, IL-6, IL-1 and IL-10 in supernatant were measured by ELISA. The BV2 cells were sub-cultured in 6-well plates (5 × 10^5^ cells/mL) and incubated with PYC (10, 25, 50 μg/mL) or DMSO (vehicle) for 1h, and then incubated with LPS (500ng/mL) for 24 h.

After treatments cell-free supernatant was collected, and TNF-α, IL-6, IL-1β, and IL-10 were measured using ELISA kits, in accordance with the manufacturer’s instructions. The absorbance at 450 nm was determined using a microplate reader. Results represent the mean ± S.D. of three experiments, with each sample examined in triplicate within each experiment.

### NF-κB luciferase assay

A total of 1 × 10^6^ BV2 cells were transfected with 2 μg NF-κB-luciferase reporter plasmids (BD Sciences, San Jose, CA, USA) using Lipofectamine in accordance with the manufacturer’s protocol (Invitrogen, #11668019, Carlsbad, CA, USA). After incubating with DNA-Lipofectamine mixtures, the cells were pre-incubated in the presence or absence of PYC for 1 h before being stimulated with LPS for an additional 6 h. After stimulation, the cells were washed, lysed and collected. Luciferase activity was measured using a Dual-Luciferase Reporter Assay System in accordance with the manufacturer’s instructions (Promega, E1910, Madison, WI, USA). Each transfection experiment was performed in triplicate and repeated at least three times.

### Electrophoretic mobility shift assay

Electrophoretic mobility shift assay (EMSA) was performed as previously described (15). AP-1 consensus oligonucleotide (5′-CGCTTGATGACTCAGCCGGAA-3′) was ^32^P-labeled with T4 polynucleotidekinase (Takara, Shiga, Japan), and then purified using Chroma-Spin TE-10 columns (Clontech, Palo Alto, CA). The cells were pre-incubated in the presence or absence of PYC for 1 h before being stimulated with LPS for 30 min. After stimulation, BV2 nuclear protein was extracted, and extracts (10 μg) were first incubated in a 25μL reaction volume for 20min at 20°C with or without unlabeled competitor oligonucleotide (100-fold molar excess). The reaction buffer consisted of 10 mM Tris HCl (pH 7.6), 50 mM KCl, 5 mM MgCl2, 1 mM DTT, 1 mMEDTA, 12.5% (vol/vol) glycerol, 0.1% Triton X-100, 8μg/mL calfthymus DNA, and 50 mM PMSF. A radiolabeled probe (50000counts/min, 1.0 ng DNA) was then added and incubated for an additional 20 min at 20°C. DNA-protein complexes were analyzed on a 5% native polyacrylamide gel at 100 V for 3 h in Tris-borate-EDTA buffer. Thereafter, the gels were dried and subjected to autoradiography.

### Statistical analyses

Data are presented as the mean ± standard deviation of at least 3 separate experiments. Statistical differences were determined by one-way ANOVA. Correction for repeated measures was performed using Dunnett or Bonferroni post-tests. P-values < 0.05 were considered statistically significant.

## Results

### PYC suppresses LPS-induced NO production in BV2 microglia in a dose-dependent manner

**Fig 1:**
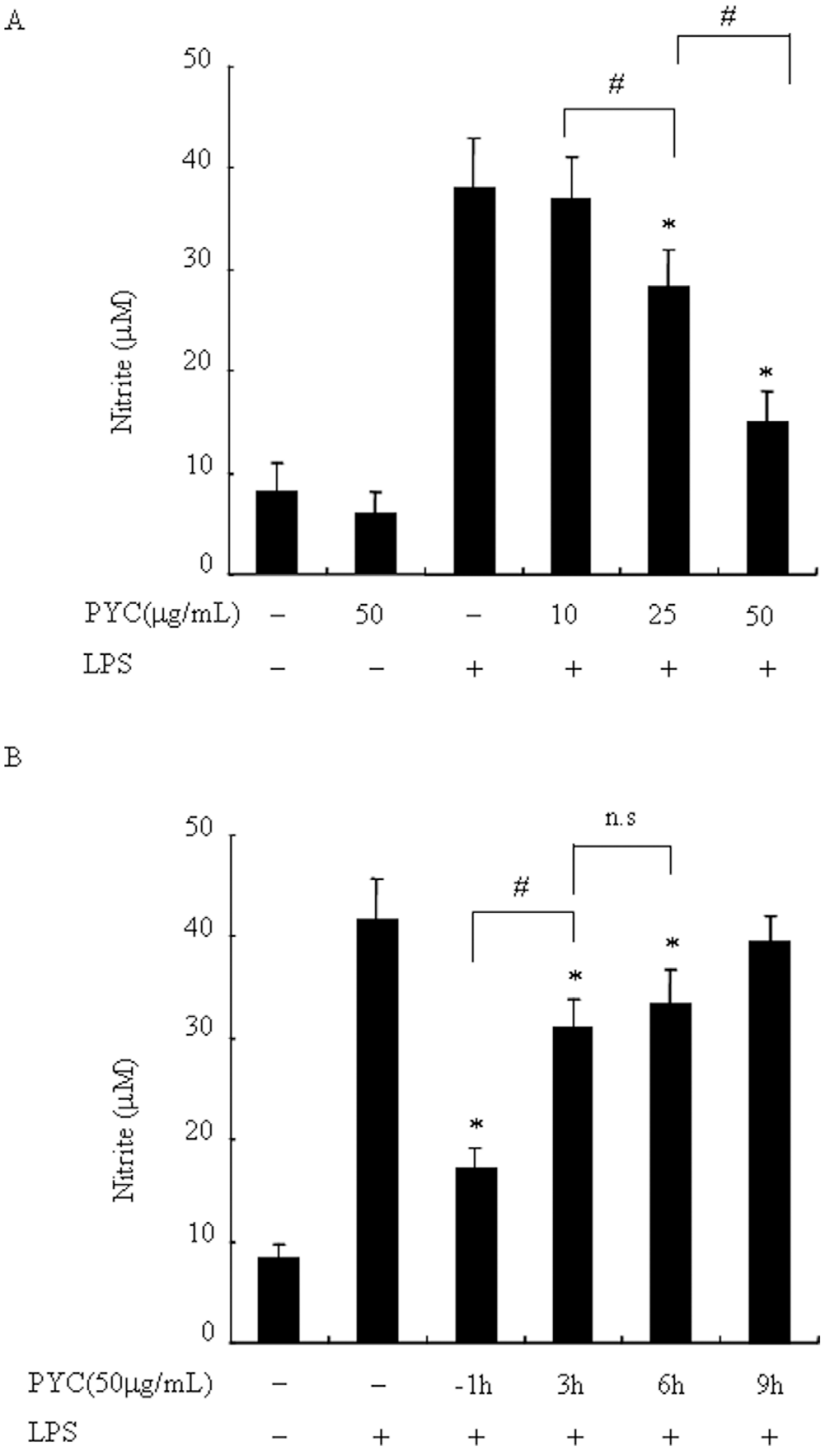
PYC suppressed LPS-induced NO production in BV2 microglia. Cells were incubated with PYC (10, 25, 50 μg/mL) or DMSO (vehicle) for 1 h before addition of LPS (500ng/mL) for 24h (A) or cells were incubated with PYC (50 μg/mL) or vehicle in indicated time before or after LPS stimulation for 24h (B). The culture supernatants were collected and analyzed for nitrite production using Griess reagent and a standard curve was created using NaNO_2_ in culture medium. Data are represented as mean ± SD from at least 3 independent experiments. *P< 0.05 compared with LPS alone. ^#^P< 0.05.

To examine substances that can suppress activation of microglial cells, we focused on studying PYC, an extract from French maritime pine bark, which has been shown to have potential anti-oxidant and anti-inflammatory effects [22]. We tested the effects of PYC on LPS-induced NO production using BV2 microglia. The amount of NO significantly increased from 8.81 ± 3.2 μM to 38.25 ± 3.5 μM 24h after LPS stimulation (P < 0.05) (Fig 1A); however, PYC pre-treatment suppressed LPS-induced NO production in a dose-dependent manner, whereas it did not affect the basal level of NO. PYC (50μg/mL) showed the highest inhibitory potential (reduced to 45% that of LPS stimulation, P < 0.05). These results indicate that PYC markedly inhibited NO production in BV2 microglial cells after LPS stimulation. Next we presumed that once microglia cells have been activated, PYC could suppress activation of microglial cells as well. We tested the effects of PYC post-treatment on NO production. After PYC post-treatment, LPS-increased NO production were reduced to 75% (3 h after LPS treatment, P < 0.05) and 80% (6 h after LPS treatment, P <0.05), when compared with levels in cells with only LPS treatment, respectively. There is no a significant decrease, when cells were treated with PYC at 9h after LPS stimulation; However, PYC pre-treatment (1 h before LPS treatment) showed the highest inhibitory potential (reduced to 45% that of LPS stimulation, P < 0.05 and P < 0.05, when compared with PYC post-treatment) (Fig 1B). Therefore, we chose PYC pre-treatment for further study.

**Fig 2:**
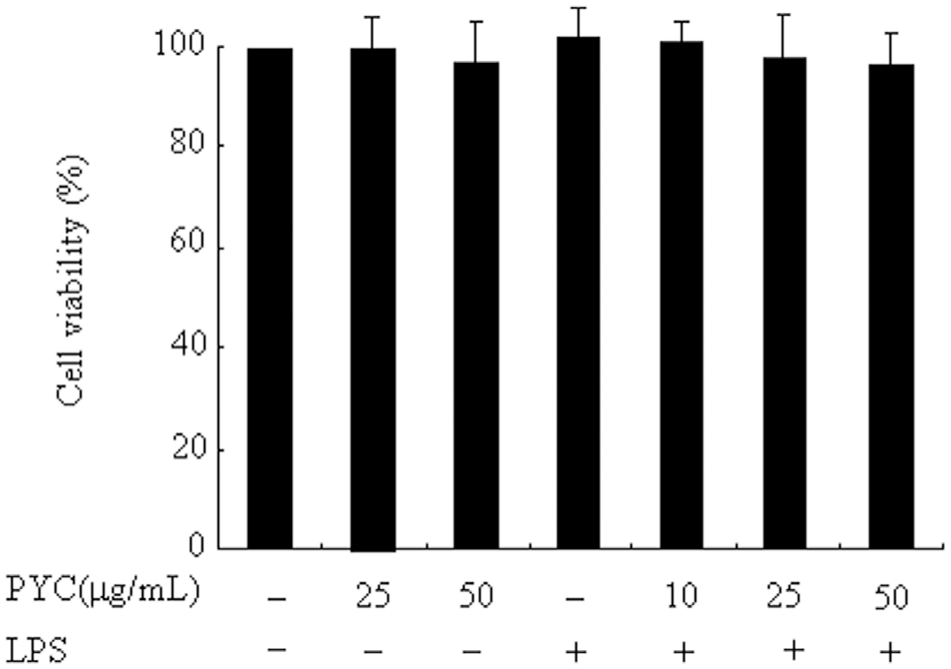
Effects of different doses of PYC on cell viability of BV2 microglia. BV2 cells were treated with PYC at various concentrations or DMSO (vehicle) for 1h. Then, LPS (500ng/mL) was added and incubated for 24h. After treatments, Cell viability was assessed by MTT and the results are expressed as the percentage of surviving cells over control cells (no addition of PYC and LPS). Data are represented as mean ± SD from at least 3 independent experiments.

It is possible that the observed inhibition of NO production was due to the cytotoxicity of PYC in LPS-stimulated BV2 microglial cells. To exclude this possibility, we performed MTT assays with BV2 microglial cells treated with PYC for 24h. PYC treatment did not change MTT activity even at the highest concentration (50 μg/mL; Fig 2). These data confirmed that the inhibition of NO production in LPS-treated BV2 microglia was not due to a PYC-induced cytotoxic reaction.

### PYC lowers LPS-induced the levels of iNOS mRNA and protein

**Fig 3:**
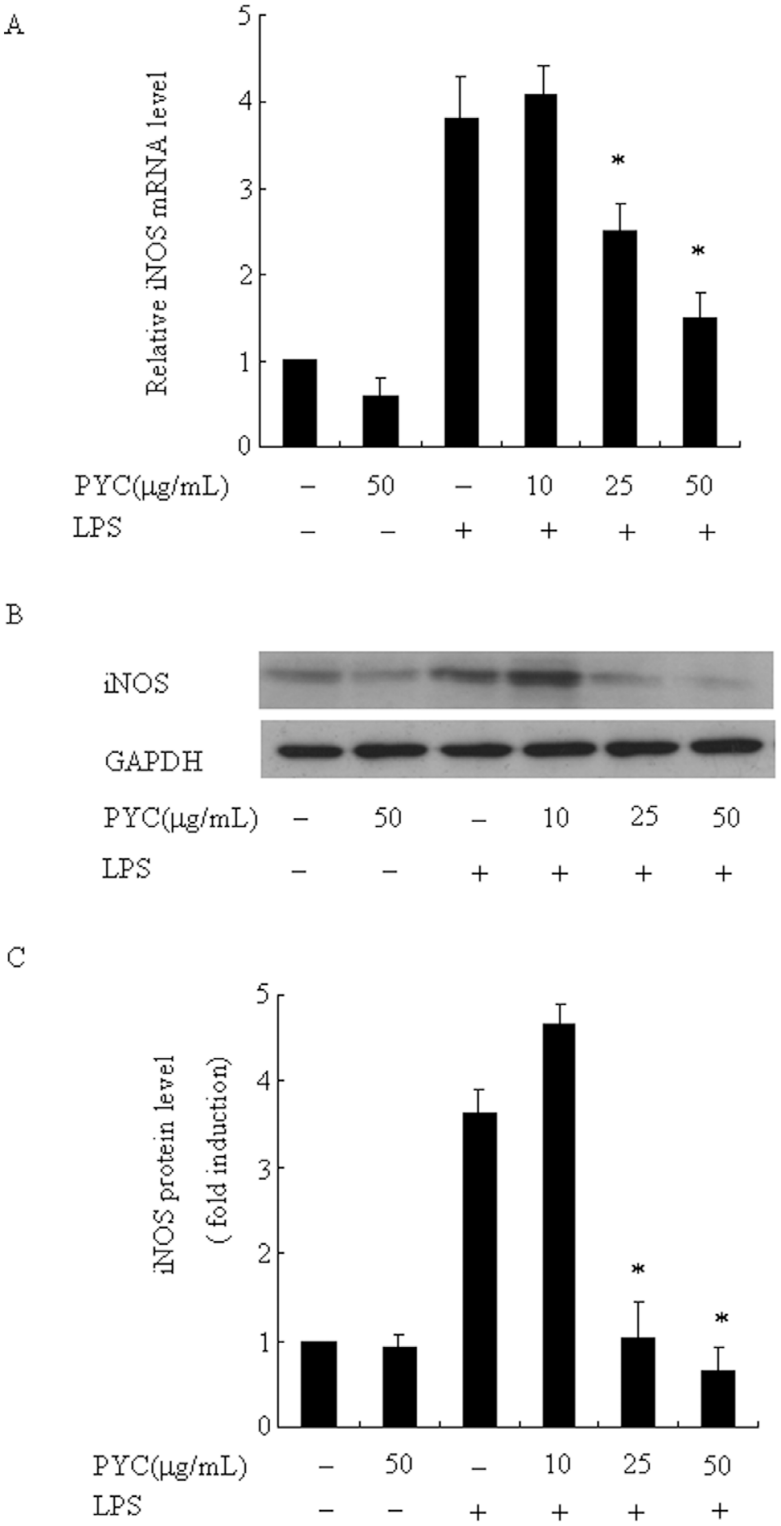
Effects of different doses of PYC on LPS-induced the levels of iNOS mRNA and protein in BV2 microglia. BV2 cells were treated with PYC at various concentrations or DMSO (vehicle) (10, 25, 50 μg/mL) for 1h. LPS (500ng/mL) was then added and incubated for an additional 24 h. Ribosomal RNAs and GAPDH were used as the total RNA or protein loading control, respectively. (A) iNOS mRNA was assessed by real-time PCR, and the mRNA level in the control (no stimuli) was arbitrarily designated as 1 for comparison. The data represent the means ± SD of 3 independent experiments. *P< 0.05 compared with LPS alone. (B) Levels of iNOS protein were assessed via Western blot. (C) Levels of iNOS protein were quantified by the NIH Image processing and analysis program. *P< 0.05 compared with LPS alone. Experiments were repeated at least 3 times and similar results were obtained.

Since NO is the product of the enzyme iNOS [23], we further tested whether inhibition of NO production by PYC is associated with a lower level of iNOS in LPS-stimulated BV2 microglial cells. Real-time PCR results showed that cells without LPS stimulation expressed a very low level of iNOS mRNA, whereas levels of iNOS mRNA were increased by 3.7-fold 24h after LPS stimulation (Fig 3A); however, after pre-treatment of PYC, increased levels were reduced to 76% and 43% in the presence of 25 μg/mL and 50μg/mL PYC, when compared with LPS treatment, respectively (Fig 3A). Consistent with the effect on the expression of iNOS mRNA, LPS also significantly enhanced the amount of iNOS protein by 3.6-fold (Fig 3B), and this upregulation was remarkably inhibited to 36% and 28% in the presence of 25 and 50μg/mL PYC, when compared with only LPS treatment, respectively (Fig 3B and 3C); however, basal levels of iNOS mRNA and protein were not changed in the presence of 50μg/mL PYC. These data indicated that the suppressive effect on NO production is at least partially through transcriptional inhibition of iNOS.

### PYC inhibits LPS-induced production of proinflammatory cytokines but not anti-inflammatory cytokines in BV2 microglia

**Fig 4:**
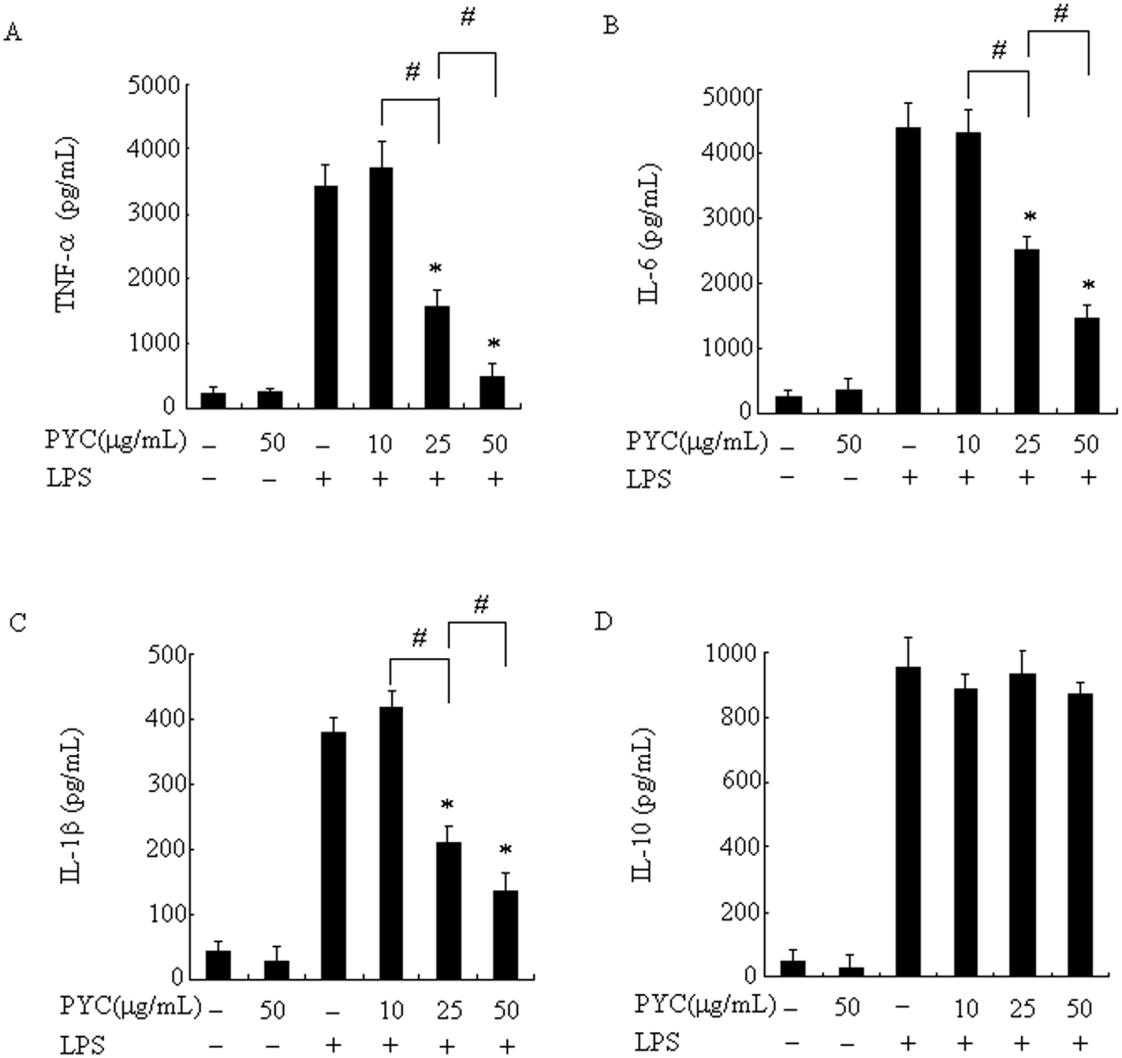
PYC suppressed LPS-induced proinflammatory cytokine production in BV2 microglia. Cells were incubated with the indicated concentrations of PYC or vehicle for 1h before LPS treatment (500ng/mL). After 24 h incubation, the culture supernatants were collected, and the amount of TNF-α, IL-6, IL-1β and IL-10 were measured by ELISA (A-D). Data are represented as mean ± SD from at least 3 independent experiments. *P< 0.05 compared with LPS alone. ^#^P< 0.05.

In the present study, we investigated the effects of PYC on LPS-induced production of the proinflammatory cytokines TNF-α, IL-6 and IL-1β and anti-inflammatory cytokine IL-10 in BV2 microglia. We found that PYC did not cause any effect on the levels of TNF-α, IL-6, IL-1β, or IL-10 in untreated cells; however, LPS treatment induced significant production of these cytokines. Pre-treatment with PYC was associated with a decrease in levels of TNF-α, IL-6 and IL-1β in a dose-dependent manner (Fig 4), and the greatest suppression was observed at 50 μg/mL PYC. The levels were inhibited to 15%, 33%, and 36% for TNF-α, IL-6, and IL-1β, respectively, when compared with those of LPS stimulation (Fig 4); however, pre-treatment with PYC did not show any effect on the production of IL-10 induced by LPS, even at the highest concentration of 50μg/mL (Fig 4D).

### PYC inhibits LPS-induced ICAM-1mRNA expression in BV2 microglia

**Fig 5:**
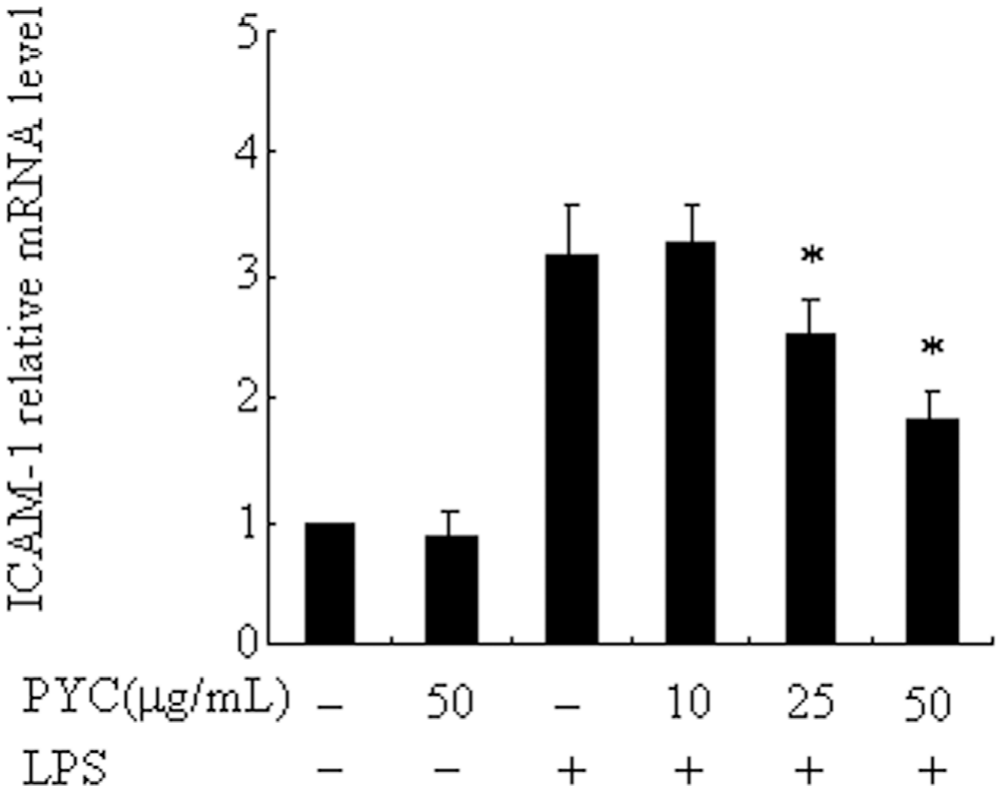
Pre-incubation of different doses PYC suppressed LPS-induced ICAM-1 expression in BV2 microglia. BV2 cells were treated with PYC or vehicle at the indicated concentrations for 1h. LPS (500ng/mL) was then added and further incubated for 24 h. Ribosomal RNAs were used as the total RNA loading control. ICAM-1 mRNA was assessed by real-time PCR, and the mRNA level in the control (no stimuli) was arbitrarily designated as 1 for comparison. The data represent the means ± SD of at least 3 independent experiments. *P< 0.05 compared with LPS alone.

ICAM-1 and its counter receptor, the lymphocyte function-associated antigen1(LFA-1), are known to be involved in inflammatory processes [5–7]. Therefore, we further investigated the effects of PYC on the expression of ICAM-1stimulated by LPS in BV2 microglial cells. The levels of ICAM-1 mRNA after stimulation with LPS were markedly lower when treated with 25 and 50μg/mLPYC, reduced to79% and 58%, separately (Fig 5); however, PYC had no effect on basal ICAM-1 expression.

### PYC inhibits LPS-induced PLIN2 expression in BV2 microglia

**Fig 6:**
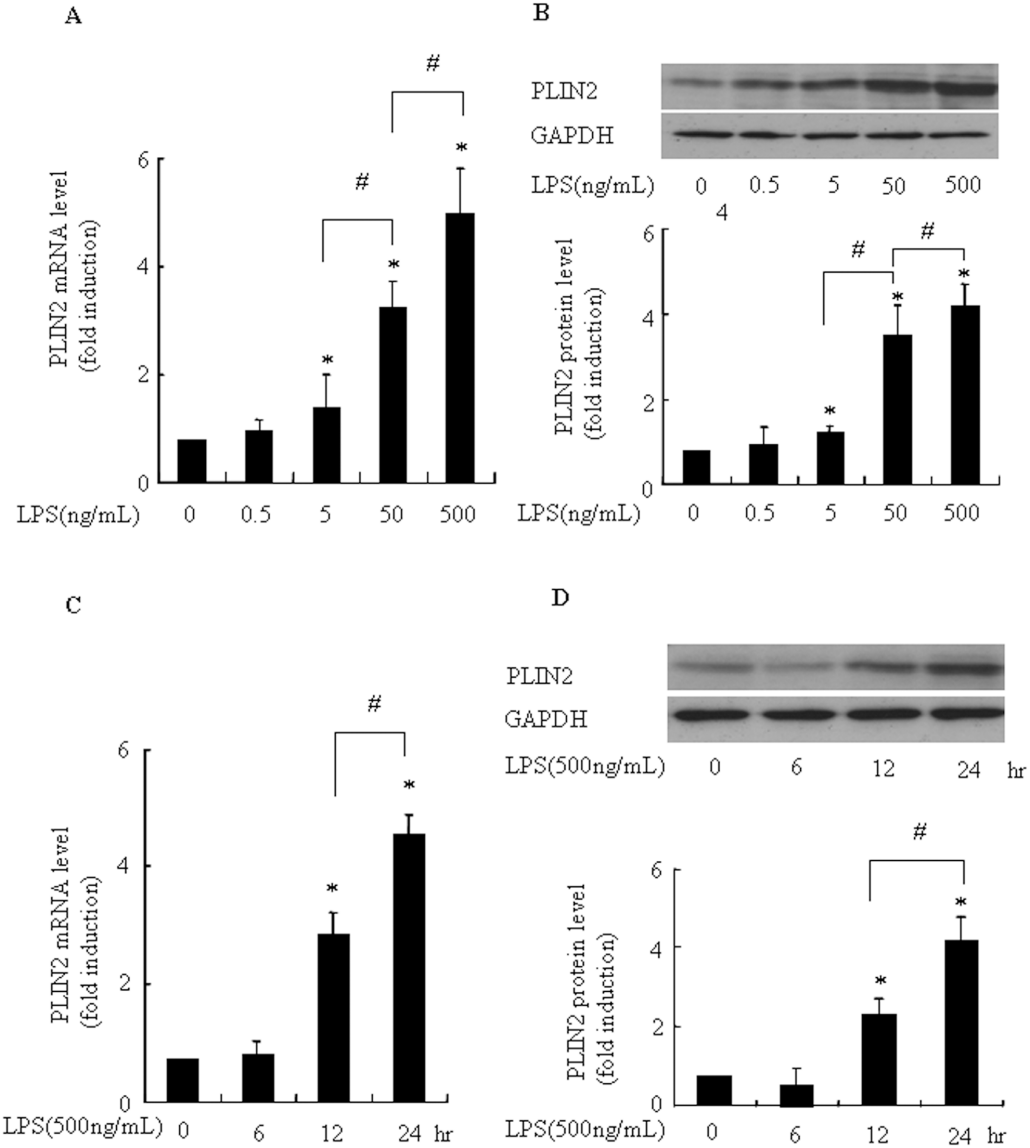
LPS increased the levels of PLIN2 mRNA and protein in a dose and time dependent manner in BV2 microglia. (A and B): LPS stimulated PLIN2 mRNA and protein expression in a dose-dependent manner. Cells were incubated with vehicle or indicated concentrations of LPS for 24 h. (C and D): LPS enhanced PLIN2 mRNA and protein expression in a time-dependent manner. Cells were incubated with vehicle or LPS (500ng/mL) for 6, 12 or 24h. Ribosomal RNAs and GAPDH were used as the total RNA or protein loading control, respectively. The PLIN2 mRNA level in the control (no stimuli) was arbitrarily designated as 1 for comparison. Levels of PLIN2 protein were quantified by the NIH Image processing and analysis program. *P< 0.05 compared with LPS alone. ^#^P< 0.05. Experiments were repeated 3 times and representative results are shown.

**Fig 7:**
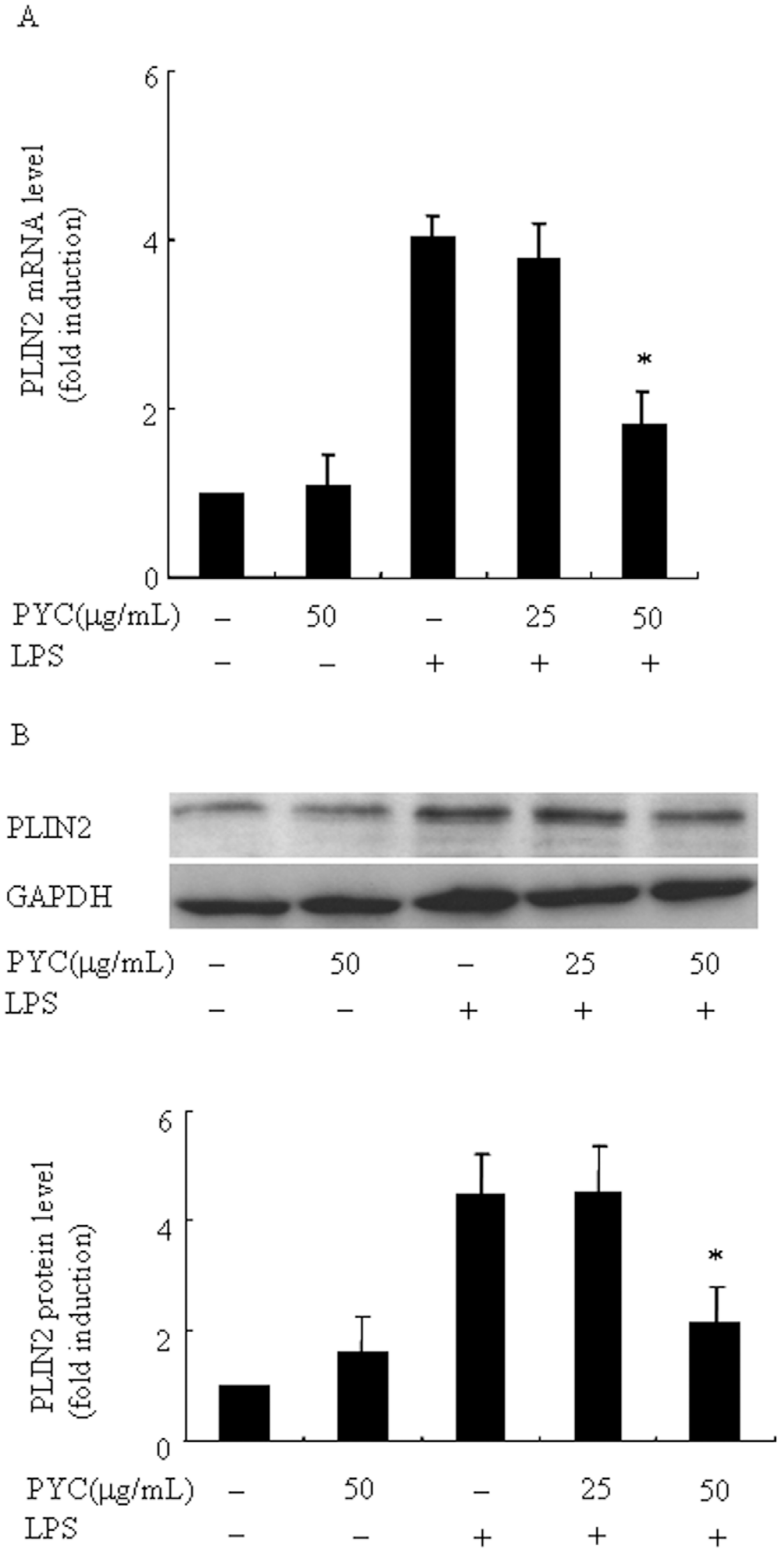
Pre-incubation of different doses PYC suppressed LPS-induced PLIN2 expression in BV2 microglia. Cells were incubated with the indicated concentrations of PYC or vehicle for 1h before 24 h LPS treatment (500ng/mL). Ribosomal RNAs and GAPDH were used as the total RNA or protein loading control, respectively. (A) PLIN2 mRNA was assessed by real-time PCR, and the mRNA level in the control (no stimuli) was arbitrarily designated as 1 for comparison. (B) Levels of PLIN2 protein were assessed via Western blot. Levels of PLIN2 protein were quantified by the NIH Image processing and analysis program. *P< 0.05 compared with LPS alone. ^#^P< 0.05. Representative data are shown. Relative mRNA and protein levels obtained from 3 independent experiments are shown in bar graphs.

**Fig 8:**
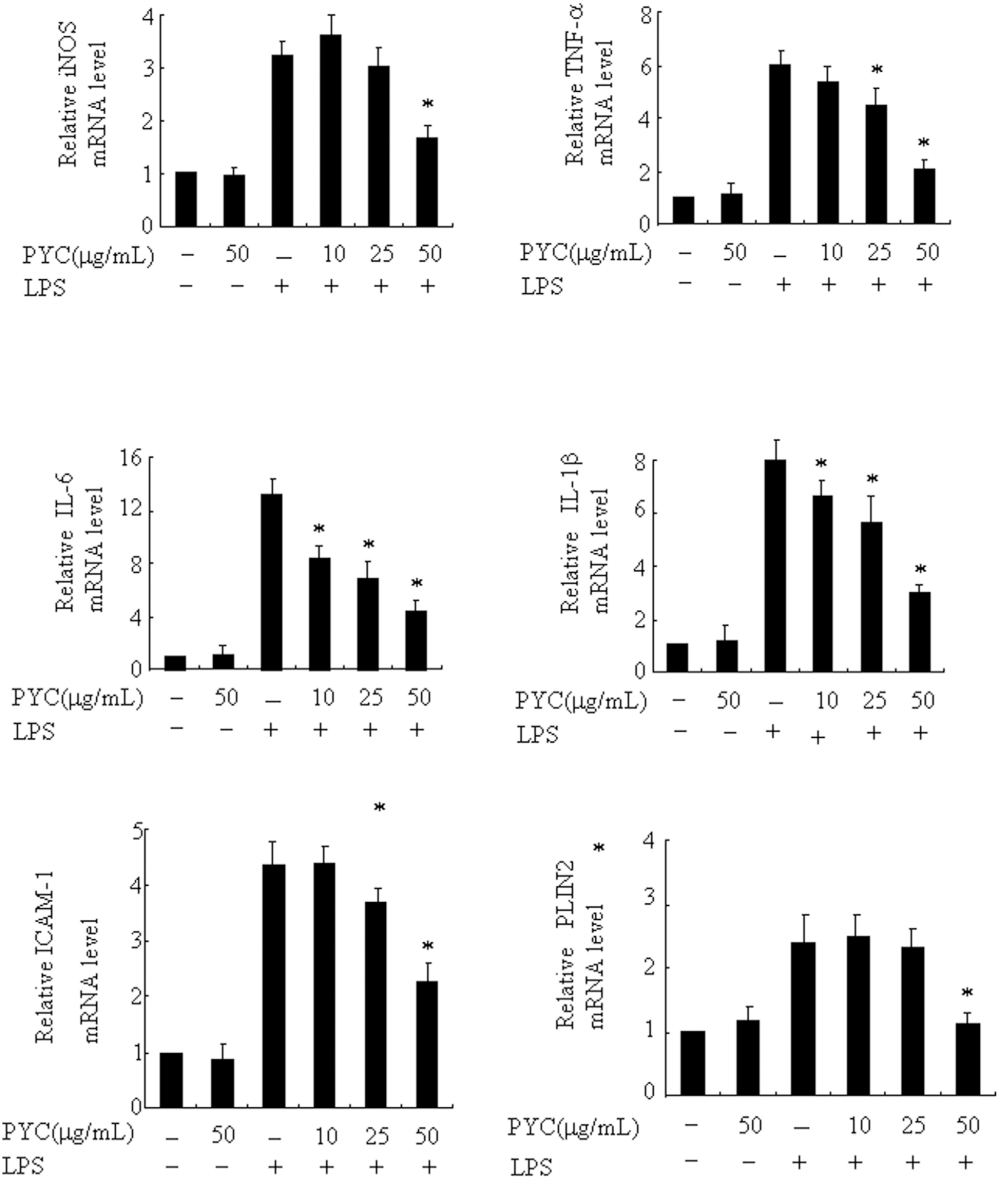
The inhibitory potential of PYC was also fully replicated in mouse primary brain microglia. Cells were treated with PYC or vehicle at the indicated concentrations for 1h. LPS (500ng/mL) was then added and further incubated for 24 h. Ribosomal RNAs were used as the total RNA loading control. mRNA levels was assessed by real-time PCR, and the mRNA level in the control (no stimuli) was arbitrarily designated as 1 for comparison. The data represent the means ± SD of at least 3 independent experiments. *P< 0.05 compared with LPS alone.

Recently, Khatchadourian et al [8] reported that PLIN2 is involved in activation of microglia and associated with the accumulation of lipid droplets, and we previously demonstrated that PYC suppressed LPS-induced PLIN2 expression in macrophages [15]. We hypothesized that PYC would inhibit LPS-induced PLIN2 expression in microglia, and then suppress activation of microglia. Therefore, in the present study, we tested the effect of PYC on PLIN2 expression in BV2 microglia. The levels of PLIN2 mRNA and protein was stimulated significantly by LPS and the effect of LPS was dose- and time-dependent (Fig 6). Twenty-four-hour incubation of 500 ng/mL LPS led to the highest expression of PLIN2. More importantly, we found that, for the first time, 50 mg/mL PYC significantly suppressed the LPS-induced increase in PLIN2 mRNA (from4.1- to 1.9-fold) and protein levels (from 4.1- to 2.1-fold) in microglia, where it did not affect basal PLIN2 expression levels (Fig 7). These results indicate that PYC suppressed PLIN2 expression, and thus PYC may contribute to the suppression of microglia activation. The inhibitory potential of PYC on activation of BV2 cells was also fully replicated in mouse primary brain microglia (Fig 8), indicating that BV2 cell line is a suitable model to study the effects of PYC on microglia.

### Inhibitory effects of PYC are mediated through NF-κB and AP-1 in LPS-stimulated BV2 microglia

**Fig 9:**
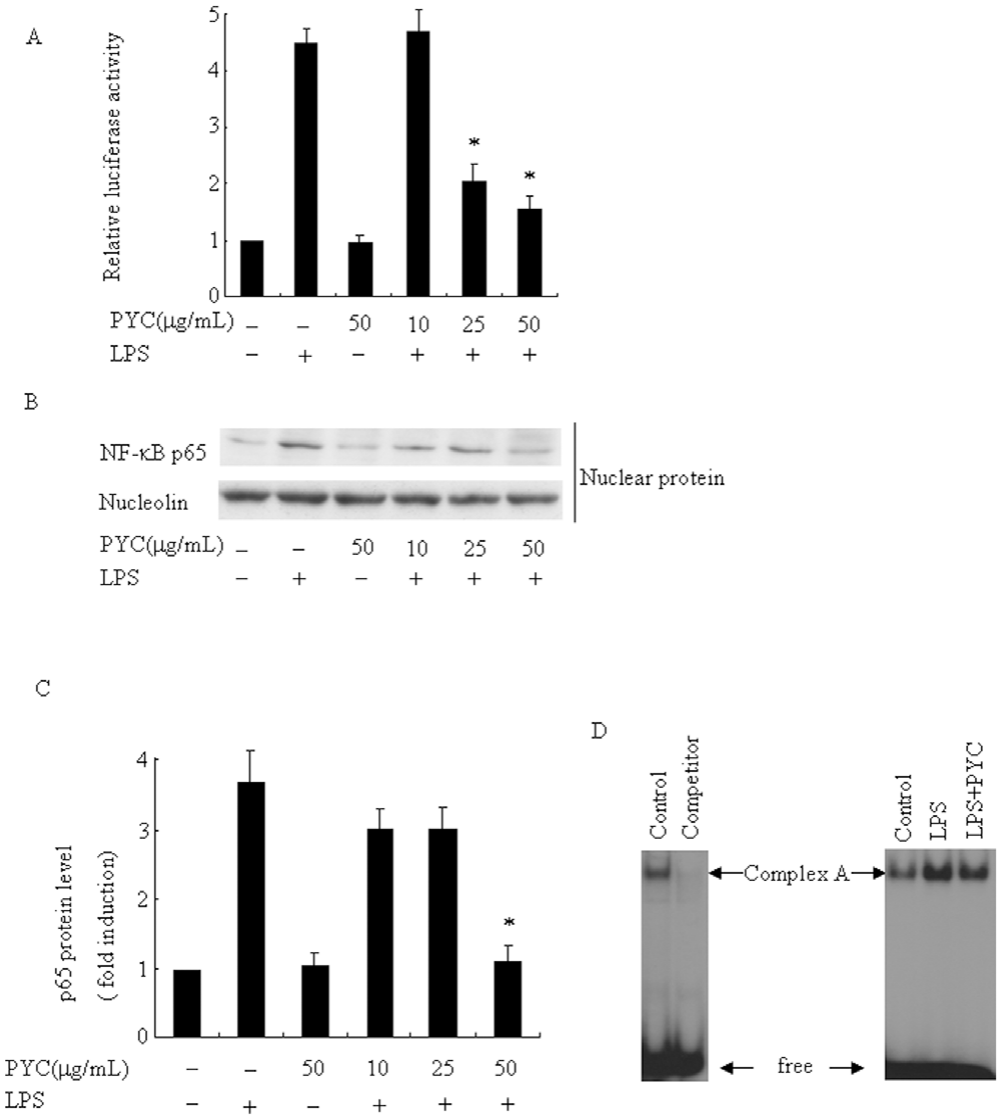
PYC suppressed LPS-induced NF-κB activity and DNA binding of AP-1 in BV2 microglia. (A) NF-κB-luciferase reporter plasmids were transfected in BV2 cells for 4 h, pre-treated with PYC 10, 25, and 50 μg/mL or DMSO (vehicle) for 1 h, then challenged with 500ng/mL LPS for 6 h. NF-κB activity was expressed as relative luciferase activity. *P< 0.05 compared with LPS alone. Cells were treated with indicated doses of PYC or DMSO (vehicle) for 1 h before 500ng/mL LPS treatment for 30 min, and then subjected to Western blot to detect p65 protein levels (B) and EMSA to assess AP-1 activation (C). ^32^P-end-labeled AP1 consensus oligonucleotide was used as a probe. Unlabeled oligonucleotide was used as a competitor. The experiment was repeated at least 3 times and similar results were obtained.

**Fig 10:**
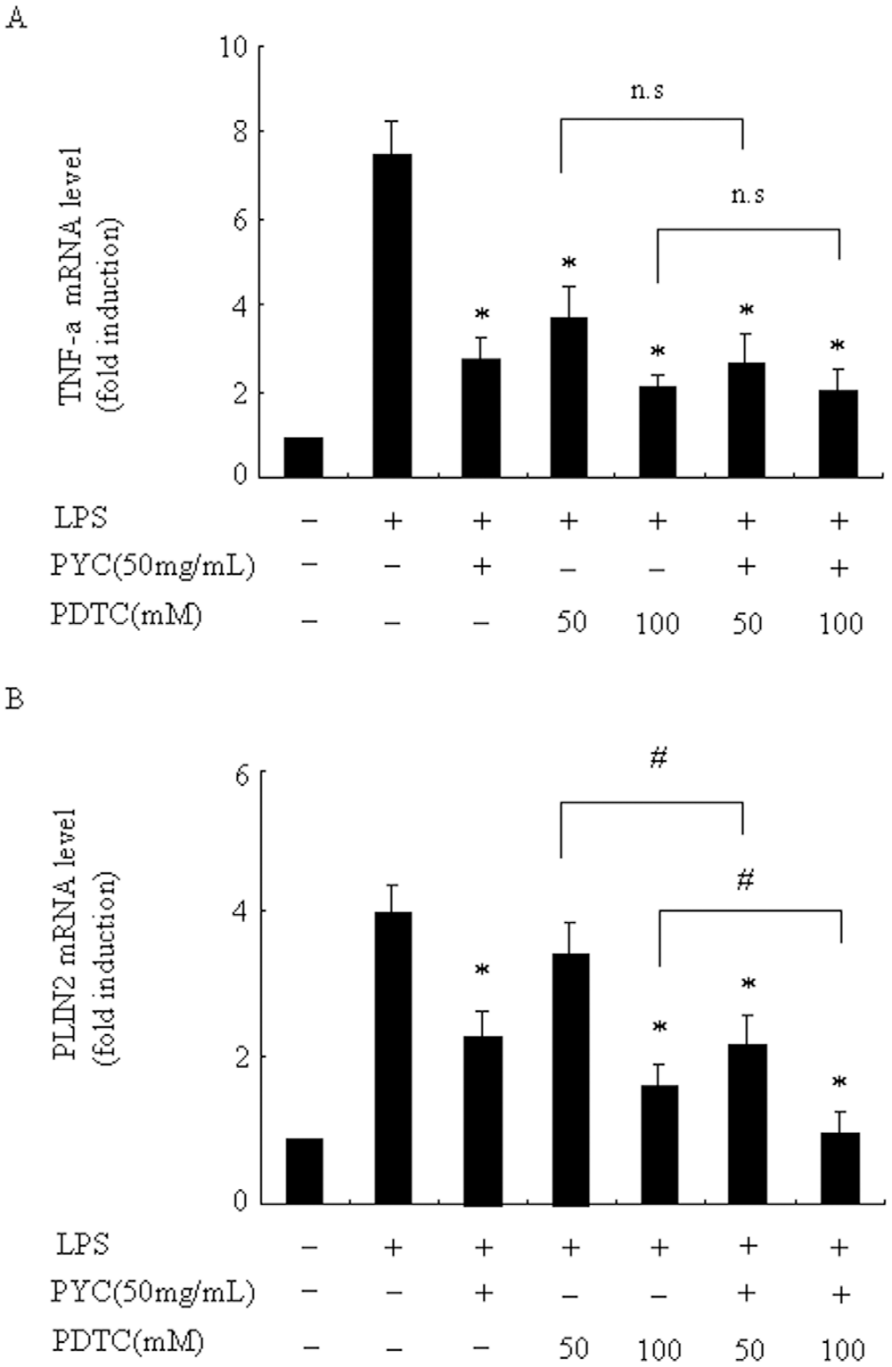
The effects of the combination of PYC and NF-κB inhibitor (PDTC) on TNF-α and PLIN2 mRNA levels. Cells were treated with PYC, PDTC (50, 100 μM) or their combination of both for 1h in LPS (500 ng/mL) was then added and further incubated for 24 h. Ribosomal RNAs were used as the total RNA loading control. mRNA level was assessed by real-time PCR, and the mRNA level in the control (no stimuli) was arbitrarily designated as 1 for comparison. The data represent the means ± SD of at least 3 independent experiments. *P< 0.05 compared with LPS alone.

Because the activation of NF-κB and AP-1 by LPS has a central role in LPS-induced production of proinflammatory cytokines, and we previously demonstrated that PYC significantly reduced LPS-induced mRNA expressions of IL-6, IL-1α, and IFN-β in RAW264.7 cells [15, 24, 25]; however, the effects of PYC on microglia is still unknown. In the present study we tested the effects of PYC on NF-κB and AP-1 activities in microglia. LPS significantly stimulated NF-κB reporter construct luciferase activity (Fig 9A). By contrast, this activity was markedly reduced to 46% and 34% of the control following 1h pre-treatment with 25 and 50 μg/mL PYC, respectively. Clearly, NF-κB p65 protein was localized within the nucleus 30 min after LPS stimulation, and PYC pre-incubation significantly attenuated the observed nuclear translocation (Fig 9B). Complex A formation was effectively inhibited by the addition of unlabeled AP—1 oligonucleotide, suggesting that complex A is formed by the AP-1 family of transcription factors (Fig 9C). LPS induced an increase in AP-1 levels, whereas this enhancement of AP-1 was clearly suppressed by pre-treatment with PYC. These results indicate that inhibition of microglial activation by PYC is at least partially via suppression of NF-κB and AP-1activities in LPS-stimulated BV2 microglial cells. Since the importance of NF-κB function in inhibition potential of PYC, we next tested whether the effects of PYC are indeed mediated by NF-κB inhibition by using NF-κB inhibitor (PDTC). We chose TNF-α as the representative of proinflammatory cytokines. As shown in Fig 10A, PDTC significantly decreased TNF-α mRNA levels induced by LPS. Since PYC and PDTC inhibited TNF-α mRNA levels to almost the same extend and the combination of PYC and PDTC did not show any synergistic effects, therefore, we presumed that PYC and PDTC might act in the same convergent pathway to suppress TNF-α mRNA levels; However, the simultaneous treatment of PYC and PDTC exhibited further suppression of PLIN2 mRNA levels than any individual treatment (Fig 10B), indicating that not only through inhibition of NF-κB activity, PYC might also inhibit PLIN2 mRNA levels though other mechanism in a NF-κB independent pathway. Further mechanisms of PYC action on the PLIN2 gene still remain to be clarified.

## Discussion

In the present study, we demonstrated for the first time that PYC significantly inhibited NO production in LPS-stimulated BV2 microglial cells. The inhibitory effects were accompanied by a decrease in iNOS expression at both the transcription and protein levels. Additionally, PYC significantly attenuated LPS-induced production of proinflammatory cytokines and adhesion molecules such as TNF-α, IL-6, IL-1β and ICAM-1. Furthermore, PYC also suppressed expression of PLIN2, whereas it did not affect production of anti-inflammatory cytokine IL-10. The mechanisms underlying these suppressive effects seem to be associated with the inhibition of NF-κB and AP-1activities.

MTT assay shows that all the concentrations of PYC used in the present study did not affect the viability of BV2 microglial cells, suggesting that the inhibitory effects of PYC on LPS-stimulated BV2 microglial cells were not due to a toxic effect. Furthermore, our results indicate that approximately more than or equal to 25μg/mL PYC could be a promising concentration for mitigating LPS-induced microglial activation.

LPS is a common inflammogen that has been used to activate microglial cells in several models both in vivo and in vitro [26]. Accumulating evidence has shown that the expression of iNOS, the key enzyme for NO production, is upregulated inactivated glial cells [27]. Additionally, pro-inflammatory cytokines such as TNF-α, IL-6, IL-1β are known to promote pro inflammatory responses mediated by microglial cells in vitro and in vivo [28, 29]. Furthermore, ICAM-1 is important in inflammatory processes in activated microglial cells [6, 7, 30]. Thus, inhibition of microglial activation and reduction of proinflammatory mediator release are a promising strategy for the prevention of neurodegenerative diseases. We focused on PYC, a natural extract of French maritime pine bark, and widely used as a dietary supplement. The main constituents are monomeric phenolic compounds (catechin, epicatechin, and taxifolin) and condensed flavonoids (procyanidins and proanthocyanidins)[22].

To the best of our knowledge, we demonstrated herein for the first time that PYC suppressed NO production induced by LPS stimulation in a dose-dependent manner. The impairment of NO production may be due to suppression of iNOS at both the transcriptional and translation levels.

We previously demonstrated that PYC suppressed the expression of IL-6, IL-1α and IFN-β genes in LPS-activated RAW264.7cells [15]. Cho etal.[31] also reported that PYC inhibited expression of IL-1β mRNA in RAW264.7 cells and IL-2 expression in JurlatE6.1 cells, a human acute T-cell leukemia cell line, through different mechanisms. In rat peritoneal mast cells, PYC inhibited TNF-α, and IL-6 protein expression and secretion [32]. Furthermore, PYC shows neuroprotective effects through antioxidant and anti-inflammatory potency, in traumatic brain injury and Parkinsonism mouse models and inhibited the upregulation of TNF-α, IL-6 and IL-1β[33, 34]. However, the effect of PYC in microglia is still unknown. In the present study, we showed that PYC significantly suppressed the production of proinflammatory cytokines, including TNF-α, IL-6 and IL-1β (Fig 4) but not IFN-β (data not shown). Based on these findings, it seems that the difference maybe due to different cell lines and culture conditions. IL-10, an anti-inflammatory cytokine, has the ability to suppress the actions of many proinflammatory cytokines and it was associated with microglial activation [35]. Therefore, we tested the effect of PYC on the production of IL-10 in LPS-stimulated BV2 cells; however, the level of IL-10 did not show any change following the treatment with PYC. ICAM-1has a crucial role in a wide range of inflammatory and immune responses [5–7, 30, 36]. It has been reported that LPS is effective in rapidly upregulating the expression of ICAM-1 [37]. Consistent with previous studies, our results showed that LPS markedly stimulated expression of ICAM-1 in BV2 microglial cells. Furthermore, this enhancement was significantly attenuated by PYC.

PLIN2 has been investigated in the context of metabolic syndrome and inflammatory diseases, especially in macrophages, adipocytes and hepatocytes [13, 14]; however, the regulation and the role of PLIN2 in microglia are still unknown. Recently, it was reported that PLIN2 is strongly expressed in macrophages and microglia at the border of organ infarcts, such as in myocardial infarction, as well as in infarcts of the liver, kidney, colon, and brain [38]. Furthermore, LPS also significantly enhanced PLIN2 expression in microglia, suggesting that PLIN2 expression could contribute to microglia-mediated inflammation in the central nervous system [38]. We previously demonstrated that PYC suppressed LPS-induced PILN2 expression in macrophages [15], however, in microglia, the effect of PYC on PLIN2 expression is still unknown. We showed for the first time that PYC clearly inhibited LPS-induced PILN2 expression both in mRNA and protein levels in microglia. This result reveals a previously unreported or unknown suppressive mechanism by PYC on microglia activation.

NF-κB and AP-1 are known to be involved in inflammatory responses in activated microglia and macrophages, and upregulates proinflammatory enzymes, cytokines and adhesion molecules, including iNOS, TNF-α, IL-6, IL-1β, ICAM-1 and PLIN2 [39–42]. Furthermore, blocking the transcriptional activity of NF-κB in the nucleus of microglial cells can suppress the expression of iNOS and proinflammatory cytokines [43, 44]. Therefore, we tested the effects of PYC on NF-κB and AP-1 activities in LPS-stimulated BV2microglial cells using the luciferase reporter, Western blot assays and Electrophoretic mobility shift assay (EMSA). Here, we clearly showed that PYC significantly reduced luciferase activity, attenuated p65 nuclear translocation, and inhibited LPS-enhanced AP-1 DNA binding. All these results indicate that downregulation of proinflammatory mediators by PYC was mediated, at least partly, via suppression of the NF-κB and AP-1 signaling pathway.

We also found that the combination of PYC and NF-κB inhibitor (PDTC) did not show any synergistic effects to suppress TNF-α mRNA levels, indicating that PYC and PDTC might act in the same converged signaling pathway to regulate TNF-α expression. However, the combination of both exhibited significantly synergistic effects to inhibit PLIN2 mRNA level, thus, we believe that not only through inhibition of NF-κB activity, PYC might inhibit PLIN2 mRNA levels though other mechanisms in NF-κB independent pathway. We previously demonstrated that PYC suppressed PLIN2 expression through post-transcriptional mechanism, facilitating degradation of PLIN2 mRNA in macrophages [20]. Many kinds of stabilizing or destabilizing factors, which bind to AU-rich stretches of 3’UTR of mRNAs, have been identified [45]. It is possible that PYC also could suppress PLIN2 expression through modification of stabilizing or destabilizing factors which regulate the PLIN2 mRNA stability. Further mechanisms of PYC action on the PLIN2 expression are still remained to be clarified in microglia.

In our previous report, we demonstrated that PYC suppressed LPS-induced enhanced activity of AP-1 in macrophages without affecting DNA binding in macrophages [15]. Interestingly, in present study, in microglia, enhancement of AP-1 DNA binding induced by LPS was attenuated by PYC. It seems that the difference may be due to different cell lines, and thus the underlying mechanism warrants investigation. In keratinocytes, PYC also inhibits ultraviolet radiation-induced NF-κB-dependent gene expression [46], and it has been shown that PYC suppressed LPS-induced IL-1β production through inhibition of NF-κB activation by abolishing IκB degradation in RAW264.7cells [31]. Anti-oxidant plant extracts, such as curcummin, may inhibit histone acetyltransferase activity of co-factors such as CREB-binding protein and p300, in turn, attenuating NF-κB activity [47, 48]. Therefore, we speculate that PYC, also as plant extract, inhibits NF-κB activity through a similar manner. This possibility is under investigation.

In summary, in the present study, we demonstrated that PYC suppressed LPS-stimulated BV2 microglial activation by inhibiting proinflammatory mediators that include iNOS, TNF-α, IL-6, IL-1β, and ICAM-1. PYC also suppressed PLIN2 expression. This is the first time that this has been demonstrated. The inhibitory potential of PYC on activation of BV2 cells was fully replicated in mouse primary brain microglia as well. Furthermore, the potent inhibition of PYC was mediated at least partially via suppression of the NF-κB and AP1 signaling pathway. Our findings indicate that PYC may be developed as a therapeutic agent for the treatment of neuroinflammatory diseases (such as Alzheimer’s disease, cerebral ischemia, and multiple sclerosis) that are characterized by excessive microglial activation. Further studies in human or mouse models are warranted.
